# A study on the prevalence of dog erythrocyte antigen 1.1 and detection of canine Babesia by polymerase chain reaction from apparently healthy dogs in a selected rural community in Zimbabwe

**DOI:** 10.4102/jsava.v87i1.1409

**Published:** 2016-10-26

**Authors:** Solomon Dhliwayo, Tariro A. Makonese, Belinda Whittall, Silvester M. Chikerema, Davies M. Pfukenyi, Musavenga T. Tivapasi

**Affiliations:** 1 Department of Clinical Veterinary Studies, University of Zimbabwe, Zimbabwe

## Abstract

A study was carried out to determine the prevalence of blood group antigen dog erythrocyte antigen (DEA) 1.1 in mixed breed dogs in rural Chinamhora, Zimbabwe. DEA 1.1 is clinically the most important canine blood group as it is the most antigenic blood type; hence, DEA 1.1 antibodies are capable of causing acute haemolytic, potentially life-threatening transfusion reactions. In this study, blood samples were collected from 100 dogs in Chinamhora, and blood typing was carried out using standardised DEA 1.1 typing strips with monoclonal anti–DEA 1.1 antibodies (Alvedia^®^ LAB DEA 1.1 test kits). Polymerase chain reaction for detecting *Babesia* spp. antigen was carried out on 58 of the samples. Of the 100 dogs, 78% were DEA 1.1 positive and 22% were DEA 1.1 negative. A significantly (*p* = 0.02) higher proportion of females (90.5%) were DEA 1.1 positive than males (69.0%). The probability of sensitisation of recipient dogs following first-time transfusion of untyped or unmatched blood was 17.2%, and an approximately 3% (2.95%) probability of an acute haemolytic reaction following a second incompatible transfusion was found. *Babesia* spp. antigen was found in 6.9% of the samples. No significant relationship (χ^2^ = 0.56, *p* = 0.45) was found between DEA 1.1 positivity and *Babesia* spp. antigen presence. Despite a low probability of haemolysis after a second incompatibility transfusion, the risk remains present and should not be ignored. Hence, where possible, blood typing for DEA 1.1 is recommended. A survey of DEA 3, 4, 5 and 7 in various breeds is also recommended.

## Introduction

Canine blood groups are genetically attributed characteristics that remain the same throughout an animal’s life (Brown & Vap 2006). They are phenotypically expressed as proteins or glycoproteins on the erythrocyte surface that is specific to a species and have the ability to elicit an immune response (Giger, Stierger & Palos 2005). Numerous blood typing nomenclatures have been postulated (Giger *et al*. 2005); however, the dog erythrocyte antigen (DEA) system has been the most commonly acceded to. Although antisera exists for only six DEAs, that is, DEA 1.1, DEA 1.2, DEA 3, DEA 4, DEA 5 and DEA 7, eight DEAs have been described, namely DEA 1.1, DEA 1.2, DEA 3, DEA 4, DEA 5, DEA 6, DEA 7 and DEA 8 (Arikan *et al*. 2009). A set of two to several alleles at one gene locus makes up a blood group system (Kohn, Classe & Weingart 2012). Apart from the DEA 1 system, a dog’s DEA blood group is either positive or negative for that blood type (Arikan *et al*. 2009). DEA 1, formerly known as A, consists of four alleles, namely negative, 1.1, 1.2, and 1.3. DEA 1.1 is inherited as an autosomal dominant trait over DEA 1.2, and the null type is recessive to both. DEA 1.1 and DEA 1.2 are the most important antigens (Giger *et al*. 2005; Goggs 2009; Van der Merwe, Jacobson & Pretorius 2002; Vap 2010). The prevalence of DEA 1.1 in the general dog population is estimated at 42% – 46% (Van der Merwe *et al*. 2002); however, prevalences of up to 80% have been recorded (Madhavan, Manju & Usha 2014).

Babesiosis is a disease of worldwide significance that causes fever, haemolytic anaemia, haemoglobinuria and death (Schoeman 2009). In humans, it is known that the blood group can have a protective effect and affect the clinical outcome of *Plasmodium falciparum* infection (Cserti & Dzik 2007; Zerihun, Degarege & Erko 2011). Owing to the similarities between malaria and canine babesiosis, the question remains whether DEA blood groups, for example, DEA 1.1, can also influence the outcome of babesiosis in endemic areas. For instance, DEA 7 was shown to have protective effects on the outcome of immune-mediated haemolytic anaemia in dogs (Miller, Hohenhaus & Hale 2004). Sterilising immunity has been demonstrated in dogs infected with *B. canis*, but only occurs in some individuals (Brandao, Hagiwara & Myiashiro 2003).

The aim of this study was to ascertain the prevalence of DEA 1.1 and *Babesia* spp. infection in dogs in a rural area, Chinamhora, Goromonzi District, Zimbabwe.

## Materials and methods

### Study area and animals

Records of native or mongrel (mixed breed) dogs from 27 resource poor villages using two dip tanks in the Chinamhora area were used. The villages were conveniently selected. The area of Chinamhora was divided into two sections, with two dip tanks, Mawu (latitude 17.5666361, longitude 31.203973) and Munyawiri (latitude 17.512737, longitude 31.141681), approximately 30 km apart being regarded as central points for each of the two sections. The Mawu dip tank section included 14 villages and blood was collected from 51 dogs, whereas the Munyawiri dip tank section included 13 villages and blood was collected from 49 dogs.

Using a protocol developed by the University of Zimbabwe veterinary teaching hospital, only clinically healthy dogs were included in the study. Briefly, a physical examination was performed that included heart and lung auscultation, temperature, pulse rate, abdominal palpation and mucus membranes evaluation. Dogs were only included in the study if the physical examination was considered normal. From this, a total of 100 dogs were selected. The ages of the dogs were classified as paediatrics (≤ 6 months), adolescents (6–18 months), adults (> 18–84 months), seniors (> 84–120 months) and geriatrics (> 120 months) according to Fortney (2012).

### Blood collection

About 2 mL of blood was collected from the cephalic vein and placed in a tube containing ethylene diamine tetra-acetic acid (EDTA) as an anticoagulant and stored at 4 °C prior to analysis. Age and sex of the dogs were recorded during blood sample collection.

## Determination of dog erythrocyte antigen

The Alvedia^®^ LAB DEA 1.1 test kits (Alvedia^®^, Alice Veterinary Diagnostic, France) were used to determine the blood group (DEA 1.1 status) according to the manufacturer’s instructions. The system is based on the migration of red blood cells on a paper strip that has previously been specially treated, under the influence of a buffer flux moving along because of capillary action. A monoclonal antibody specific to the DEA 1.1 antigen has been incorporated on a 1 mm length in the strip. This antibody will retain positive DEA 1.1 red blood cells. It is characterised by the presence of a red band on the mid-portion of the strip (in front of ‘DEA 1.1’ as written on the kit). When the test is negative, the red control band, located on the upper part of the strip (written ‘C’ on the kit), has to appear, ensuring the test has run successfully. If not, then the test must be repeated (http://www.alvedia.com).

### *Babesia* spp. antigen detection using polymerase chain reaction

Of the EDTA samples collected, 29 female and 29 male dog samples were chosen at random and then subjected to DNA extraction and subsequent polymerase chain reaction (PCR) amplification for *Babesia* marker. Genomic DNA extraction was done using a Qiagen DNA extraction or purification flexigene^®^ kit following the manufacturer’s instructions (https://www.qiagen.com). PCR targeting the 18s ribosomal RNA subunit of *Babesia* antigen was done using reverse line blot (RLB) primers (Inqaba Biotechnical Industries, South Africa) F2 (5’-GAC ACA GGG AGG TAG TGA CAA G-3’) and RLB-R2 (biotin-5’-CTA AGA ATT TCA CCT CTG ACA GT-3’) with a size of 450–560 base pairs (Gubbels *et al*. 1999). Briefly, 5 µL of extracted DNA was added to 0.5 µL of each primer (10 µM) and 10 µL of Dreamtaq^®^ PCR Master Mix and topped up to 25 µL with deionised water. The conditions for PCR included an initial denaturation step at 95 °C for 3 min, followed by 35 cycles of denaturation at 95 °C for 30 s, annealing at 57 °C for 30 s and extension at 72 °C for 60 s. Final extension was done at 72 °C for 7 min. PCR was performed on a Perkin–Elmer 2400 thermal cycler (Perkin–Elmer Applied Biosystems, Foster City, USA). The PCR products were then analysed in a 1.5% agarose gel prepared in TBE 1X (pH 8.3) and stained with ethidium bromide to a final concentration of 0.5 µg/mL. Roche molecular weight maker VIII or XIV was loaded into the first and last wells of the gel. Eight microlitres of the sample was loaded in the wells in between. Electrophoresis was run at a constant voltage of 120V in TBE 1X. After 1 h of electrophoresis, the gels were viewed on a UV Transilluminator.

### Statistical analysis

Statistical analysis was performed using R-statistical programme (R Core Team 2013, R Foundation for Statistical Computing, Vienna, Austria). A database including age, sex, DEA 1.1 status and *Babesia* infection status was created in Microsoft Excel. The prevalence of DEA 1.1 and *Babesia* infection was determined as the number of positives divided by the total number of samples. The probability of a dog becoming sensitised from first-time transfusion of blood that was not typed or cross-matched was calculated using the following formula: % DEA 1.1 negative × % DEA 1.1 positive/100 (Ferreira, Gopegui & Matos 2011). The probability of the same dog developing an acute haemolytic reaction with a second incompatible transfusion using untyped blood from any other dog was calculated using the formula: %DEA 1.1 negative × % DEA 1.1 positive × % sensitisation for the first transfusion/10 000 (Ferreira *et al*. 2011). For univariable analysis, the DEA 1.1 status of the dog was used as the outcome variable with sex, age and the presence of *Babesia* antigen as the predictor variables. Chi-squared tests were used to compare the significance of the association between the outcome and predictor variables. A two sample *t*-test was used to compare the mean ages between DEA 1.1 positive and DEA 1.1 negative dogs. Only outcomes with a *p* < 0.05 were considered as significant.

## Ethical considerations

Ethical approval for use of dogs and for all protocols in this study was obtained from the Ethical and the Higher Degrees committees of the Faculty of Veterinary Science, University of Zimbabwe. The purpose of this study was well explained to the owners of the dogs attending two dip tanks in the studied area, who all expressed consent to participate in the study. Standard operating procedures were followed for collection of blood samples. It was ensured that dogs were subjected to minimal, pain-free handling during blood collection.

## Results

**FIGURE 1:**
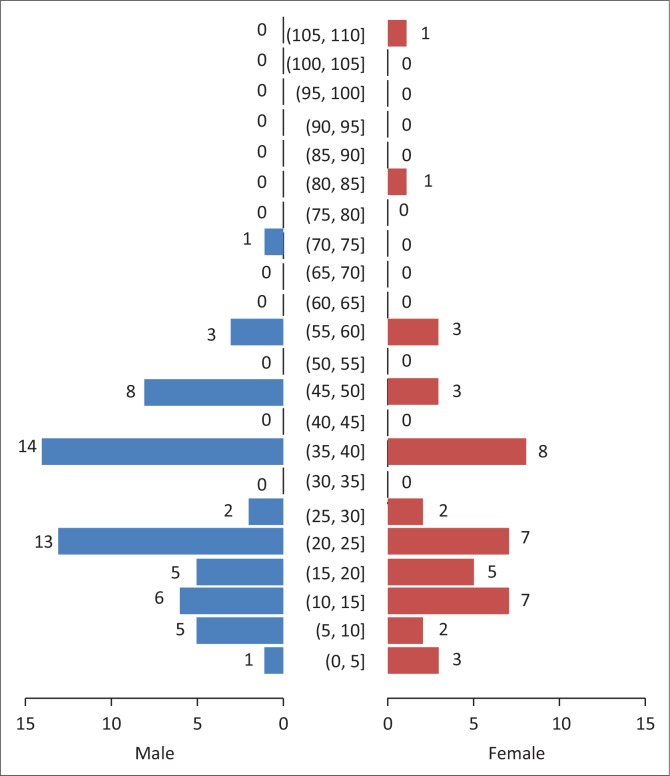
Population pyramid showing the age of dogs in months, with males and females separated.

**FIGURE 2:**
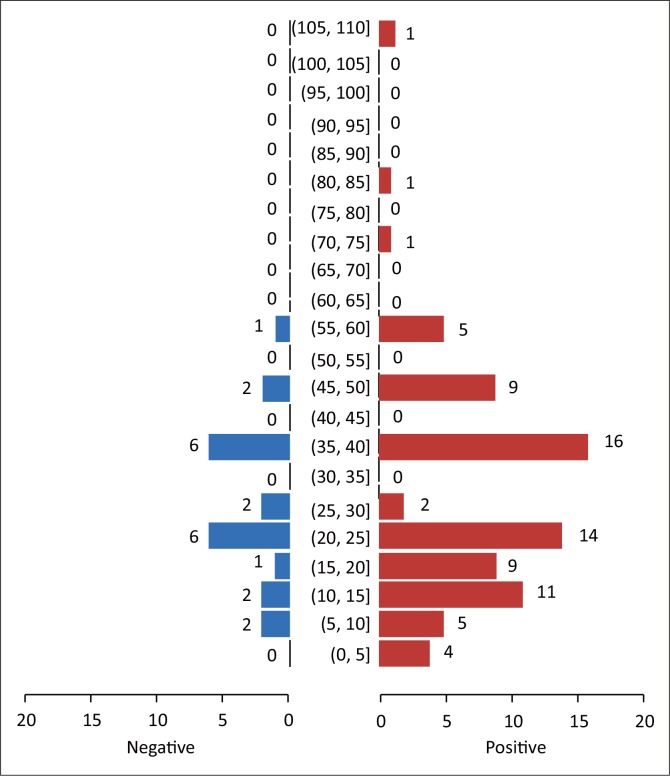
Population pyramid showing the age in months according to dog erythrocyte antigen 1.1 status in dogs sampled from Chinamhora.

The age distribution of the 100 sampled dogs is shown in Figure 1. In summary, the ages were skewed to the left with respective mean and median ages of 29.6 months and 24 months. The age range was 4–108 months, and 54% of the dogs were ≤ 2 years old. Overall, the prevalence of DEA 1.1 positive dogs was 78% (78/100; 95% confidence interval [CI]: 68.6% – 85.7%). There was no significant difference (*p* > 0.05) of DEA 1.1 positivity according to location (Mawu 76.5%, Munyawiri 79.6%). However, female dogs (90.5%) had a significantly (*p* = 0.02) higher DEA 1.1 positivity than male dogs (69.0%) (Table 1). No significant difference was noted between the mean age of DEA 1.1 positive dogs (29.8 months) and that of negative ones (28.9 months) (Figure 2). The probability of a recipient dog becoming sensitised following first-time transfusion of untyped or unmatched blood was 17.2%. An approximate 3% (2.95%) probability of an acute haemolytic reaction following a second incompatible transfusion was found. *Babesia* spp. antigens were amplified only in four dogs (6.9%, 4/58) (Table 2). There was no significant association between DEA 1.1 positivity and the presence of *Babesia* spp. antigen (χ^2^ = 0.56, *p* = 0.45).

**TABLE 1:** Prevalence distribution of dog erythrocyte antigen 1.1 according to sex in dogs sampled from Chinamhora.

Sex	Number tested (*n*)	DEA 1.1 status			
		Positive		Negative	
		*n*	%	*n*	%
Male	58	40	69.0	18	31.0
Female	42	38	90.5	4	9.5
**Overall**	**100**	**78**	**-**	**22**	**-**

DEA, dog erythrocyte antigen.

**TABLE 2:** Prevalence distribution of dog erythrocyte antigen 1.1 and *Babesia* antigen in dogs sampled from Chinamhora.

DEA 1.1 blood type	*Babesia* antigen		
	Positive	Negative	Total
Positive	2	43	45
Negative	2	11	13
**Total**	**4**	**54**	**58**

DEA, dog erythrocyte antigen.

## Discussion

This study investigated the prevalence of DEA 1.1 in dogs from Chinamhora, Goromonzi district in Zimbabwe using a laboratory test previously reported to be accurate (Giger *et al*. 2005). The test has a high sensitivity (88%) and specificity (100%) (http://www.alvedia.com), thus reducing the possibility of false negative and false positive reactions.

The respective lowest and highest type DEA 1.1 positivity frequencies have been reported as 29% (Wriensendorp, Albert & Tempelton 1976) and 96.7% (Bedrica *et al*. 2004). In this study, the prevalence of DEA 1.1 positive dogs (78%) was more than two and a half times the lowest result previously reported. The expression of DEA 1.1 in the studied mixed breed dog population was higher when compared with previous studies performed elsewhere (Esteves *et al*. 2011; Giger *et al*. 2005; Van der Merwe *et al*. 2002). Ferreira *et al*. (2011) also reported a higher frequency of DEA 1.1 expression in mixed breed dogs in contrast to earlier reports of lower frequencies of this antigen in mixed breed dogs (Novais, Santana & Vicentin 1999; Wriesendorp *et al*. 1976). However, the prevalence was similar to that found in a study done by Madhavan *et al*. (2014), who recorded a prevalence of 80% in southern India. It has been suggested that the frequency of DEA 1.1 expression might differ depending on geographic variations and breed (Arikan *et al*. 2009; Esteves *et al*. 2011). Unlike in other studies (Esteves *et al*. 2011; Ferreira *et al*. 2011), sex had an effect on the prevalence of DEA1.1 in the current study. The significantly higher DEA 1.1 expression in female compared to male dogs observed in this study is difficult to explain. Considering the small size of the present survey, more studies are needed on a larger scale to determine the role of sex and breed on the frequency of DEA 1.1 in the country.

In practice, DEA 1.1 negative dogs are considered the preferred donors (Hohenhaus 2004). The observed relatively low proportion of dogs that are DEA 1.1 negative makes the search for donors difficult to accomplish in the studied population. The risk for sensitisation after first-time transfusion of untyped blood was found to be considerable but lower than that reported earlier from elsewhere (Ekiz *et al*. 2011; Ferreira *et al*. 2011). The probability of an acute haemolytic reaction after a second incompatible transfusion was also found to be lower than that reported in previous studies (Ekiz *et al*. 2011; Ferreira *et al*. 2011).

The best method of evaluating the presence of *Babesia* spp. antigen in apparently healthy animals is using PCR (Costa-Junior *et al*. 2012). In the apparently healthy dogs in this study, the prevalence of *Babesia* spp. antigen was low, and this agrees with earlier observations (Adaszek, Martinez & Winiarczyk 2011). However, the *Babesia* spp. present in the studied dogs were not identified. Blood types could be related to some diseases. DEA 7 has been reported to be associated with a significant protective effect in Cocker Spaniels with immune-mediated haemolytic anaemia (Miller *et al*. 2004). This study failed to demonstrate a significant association between DEA 1.1 positivity and the presence of *Babesia* spp. antigen. However, the small sample size of *Babesia* spp. antigen-positive dogs could have cofounded the association with DEA 1.1 positivity. Further studies are required to determine the *Babesia* spp. present in dogs in the country and also to assess the role of blood type in babesiosis.

In conclusion, this study is the first to show the prevalence of blood type DEA 1.1 in a mixed breed rural dog population in Zimbabwe. DEA 1.1 frequency was high, whereas the presence of *Babesia* spp. antigen was low. Despite a low probability of haemolysis after a second incompatibility transfusion, the risk exists and should not be ignored. Hence, where possible, blood typing for DEA 1.1 is recommended.
